# SARS-CoV-2 UK, South African and Brazilian Variants in Karachi- Pakistan

**DOI:** 10.3389/fmolb.2021.724208

**Published:** 2021-10-25

**Authors:** Adnan Khan, Muhammad Hanif, Akhtar Ahmed, Sarosh Syed, Saqib Ghazali, Rafiq Khanani

**Affiliations:** ^1^ Karachi Institute of Radiotherapy and Nuclear Medicine (KIRAN), Karachi, Pakistan; ^2^ Department of Molecular Pathology, Hashmanis Group of Hospitals, Karachi, Pakistan; ^3^ Advanced Laboratories, Karachi, Pakistan; ^4^ Citilab Diagnostic Center, Karachi, Pakistan; ^5^ Global Research and Reference Labs, Karachi, Pakistan

**Keywords:** COVID-19, RT-qPCR, pandemic, variants of concern, SARS-CoV-2 (2019-nCoV)

## Abstract

The COVID-19 pandemic has been evolving in Pakistan with the emergence of the United Kingdom, South African, and Brazilian variants. These variants of concern (VOC) are known for increased transmissibility and can also be responsible for avoiding immune responses. The gold standard to detect VOC is sequencing, however routine genomic surveillance in resource-limited countries like Pakistan is not always readily available. The inadvertent detection of the B.1.1.7 (United Kingdom) VOC by a target failure due to the key deletion in spike Δ69-70 by commercially available PCR assay helps to understand target failures as an alternative approach to detect variants. In pursuit of VOC it was further discovered that a deletion in the ORF1a gene (ORF1a Δ3675-3677) is common in B.1.1.7, B.1.351 (South African), and P.1 (Brazilian) VOC. The Real-Time Quantitative PCR (RT-qPCR) assay can distinguish target failures and can discriminate SARS-CoV-2 VOC. The study uses positive samples archived in respective labs. Samples were divided into two groups. Group I constitutes 261 positive samples out of total of 16,964 (1.53%) performed from August till September 2020, while group II consists of 3501 positive samples out of a total of 46,041 (7.60%) performed, from November 2020 till January 2021. The RT-qPCR analysis showed that no VOC was present in positive samples of group I. However, a staggering difference in results was noted in group II where the positivity ratio increased exponentially and the VOC started appearing in significant numbers (53.64%). This concludes that the third wave in Pakistan is due to the importation of SARS-CoV-2 variants.

## Introduction

The global outbreak of the severe acute respiratory syndrome coronavirus-2 (SARS-CoV-2), the causative agent of COVID-19 disease, has ravaged the world by surprise. As of March 28, 2021, SARS-CoV-2 related infections have surpassed over 128,247,719, with 2,804,719 deaths globally. In Pakistan, the virus has affected more than 659,116 individuals, with more than 14,256 reported deaths to date (www.covid.gov.pk/stats/pakistan) (access date; 29-3-2021). SARS-CoV-2 has been responsible for overwhelmed health care systems, coerced shutting down of schools and gatherings, and pulling the planet into an economic recession. Despite the fact that 2020 was a challenging year, 2021 seems like it will not be easy either with the surfacing of multiple variants of SARS-CoV-2.

One of the notorious variants of SARS-CoV-2 characterized as lineage B.1.1.7 became known in the United Kingdom in December 2020 (www.gov.uk/government/publications/investigation-of-novel-sars-cov-2-variant-variant-of-concern-20201201). Spreading like a wildfire, the UK variant has reported to be rapidly expanding to 101 countries as of March 18, 2021 (www.gisaid.org/hcov19-variants/). A similar pattern of staggering rise in SARS-CoV-2 related infections was observed in South Africa mainly due to the emergence of lineage B.1.351 (Hsiao et al., 2020). The increased transmission rates of both these variants are attributed to the mutation in the receptor binding site of spike protein (Gu et al., 2020). The South African variant presents a possible immune escape to antibodies by having two additional mutations in the spike protein (E484K and K417N) (Wibmer et al., 2021). Moreover, It was reported in a concerning development that a different set of mutations (N501Y, E484K, and K417T) started surfacing in a new P.1 variant of SARS-CoV-2 in Brazil (Faria et al., 2021).

The first case of Covid-19 in Pakistan was reported on Feb 26, 2020, which was the beginning of the arbitrary first wave that reached its peak on June 14, with 7,000 cases per day, and went down to as low as 264 cases on August 29, 2020. The trough was maintained till Oct 14, 2020, which hallmarked the beginning of the so-called second wave. The peak of the second wave reached on December 7 with 3,795 reported new infections followed by a trough on February 16, 2021, with 747 new cases per day. However it was not a true trough and this decline was followed by a gradual rise in daily new cases arbitrarily labeled as the third wave with its new spike of 4,767 cases on March 28, (Worldometer, 2021) (www.worldometers.info/coronavirus/country/pakistan/).

The current emergence of SARS-CoV-2 variants of concern (VOC) following the phase of genetic stability of the virus is a matter of great concern since numerous new escape variants could surface in the future and can lead to epidemic resurgence, as seen in other countries. An increase in transmission of the virus augments chances for the emergence of SARS-CoV-2 VOC. While sequencing is the gold standard, it cannot always be scaled right away in limited-resource countries. Therefore a multiplexed RT-qPCR assay to detect VOC is used here. The assay can be helpful in rapid mapping VOC in countries like Pakistan.

## Materials and Methods

### Ethics

The study was approved by the Ethical Review Committee of Karachi Institute of Radiotherapy and Nuclear Medicine (KIRAN), Karachi, Pakistan.

### Sampling of SARS-CoV-2

Nasopharyngeal and oropharyngeal specimens were collected for testing of SARS-CoV-2 by reverse transcription (RT) polymerase chain reaction (RT-qPCR). This was achieved in the Molecular pathology section of Advanced Laboratories, Hashmanis Laboratories, and Citilab Diagnostic Centers in Karachi. The samples which came positive were archived in respective labs and were used in the present study for variant detection. Briefly, positive samples were extracted using a viral Nucleic acid kit (Systaac, United States) according to manufacturers’ instructions. The extracts were then subjected to RT-qPCR. The samples before extraction were divided into two categories, samples collected and archived from August to October England, 2020 (group I) and the samples collected from November 2020 to January GISAID, 2021 (group II) respectively.

### Primers/Probes

Three primers/probes set CDC_N1 (forward primer 5’-GAC​CCC​AAA​ATC​AGC​GAA​AT-3’, reverse primer 5’-TCT​GGT​TAC​TGC​CAG​TTG​AAT​CTG-3’, probe FAM-ACCCCGCATTACGTTTGGTGGACC-BHQ1), ∆69/70 del (forward primer 5’-TCA​ACT​CAG​GAC​TTG​TTC​TTA​CCT-3’, reverse primer 5-TGG​TAG​GAC​AGG​GTT​ATC​AAA​C-3’, probe HEX-TTCCATGCTATACATGTCTCTGGGA-BHQ1), and ∆ORF1a-del (forward primer 5’-TGC​CTG​CTA​GTT​GGG​TGA​TG-3’, reverse primer 5’-TGC​TGT​CAT​AAG​GAT​TAG​TAA​CAC​T-3’ and probe Cy5-GTTTGTCTGGTTTTAAGCTAAAAGACTGTG-BHQ2) were used in the assay to detect VOC by targeting the Δ3675-3677 SGF deletion in the ORF1a gene (not been reported in other SARS-CoV-2 lineages) and by targeting the Δ69/70 HV deletion in the spike gene (nucleocapsid (N1) gene on FAM, spike ∆69–70 deletion on HEX, and ORF 1a ∆3675–3766 deletion of Cy5 fluorophores respectively). The 100 µM stock solutions of primers/probes were prepared for further processing.

### Multiplex RT-qPCR

The multiplex RT-qPCR assay used here was reported earlier by Vogels et al. (2021). Briefly, for RT-qPCR reaction a probe qPCR master mix along with hot start RT enzyme mix (Thermo Scientific) is used. For qPCR reaction 20 µM of working stocks of primers/probes mix was prepared using 100 µM stocks. 10 µl of universal probe mix, 1 µl of hot-start RT enzyme mix, and 4 µl of nuclease-free water was added in 4 µl of primers/probe mix. After preparation of the mix, the thermal cycler was set for FAM, HEX, and Cy5 fluorophores while the thermal cycling conditions were slightly modified for optimized results. Thermal cycler conditions were single-step reverse transcription at 50°C for 20 min, initial denaturation at 95°C for 15 min, followed by 42 cycles of denaturation at 95°C sec for 12 s, annealing at 55°C for 30 s, and the plate read.

### Interpretation

All positive samples, subjected to the above-mentioned RT-qPCR assay were analyzed and interpreted as exhibited in Table 1.

**TABLE 1:** Interpretation of results of RT-qPCR assay for SARS-CoV-2 variants.

Interpretation	CDC_N1	∆69/70del	∆3675–3766 del
Potentially B.1.1.7	CT ≤ 35	Undetected	Undetected
Potentially B.1351 or P.1	CT ≤ 35	CT ≤ 35	Undetected
Potentially B.1375	CT ≤ 35	Undetected	CT ≤ 35
Other lineages	CT ≤ 35	CT ≤ 35	CT ≤ 35

### Statistical Analysis

Continuous variables were compared using Student’s *t* test. Fisher’s exact test was used to analyze the difference of gender in SARS-CoV-2 VOC infection among group I and group II. All statistical analysis was carried out using SPSS.

## Results

**FIGURE 1:**
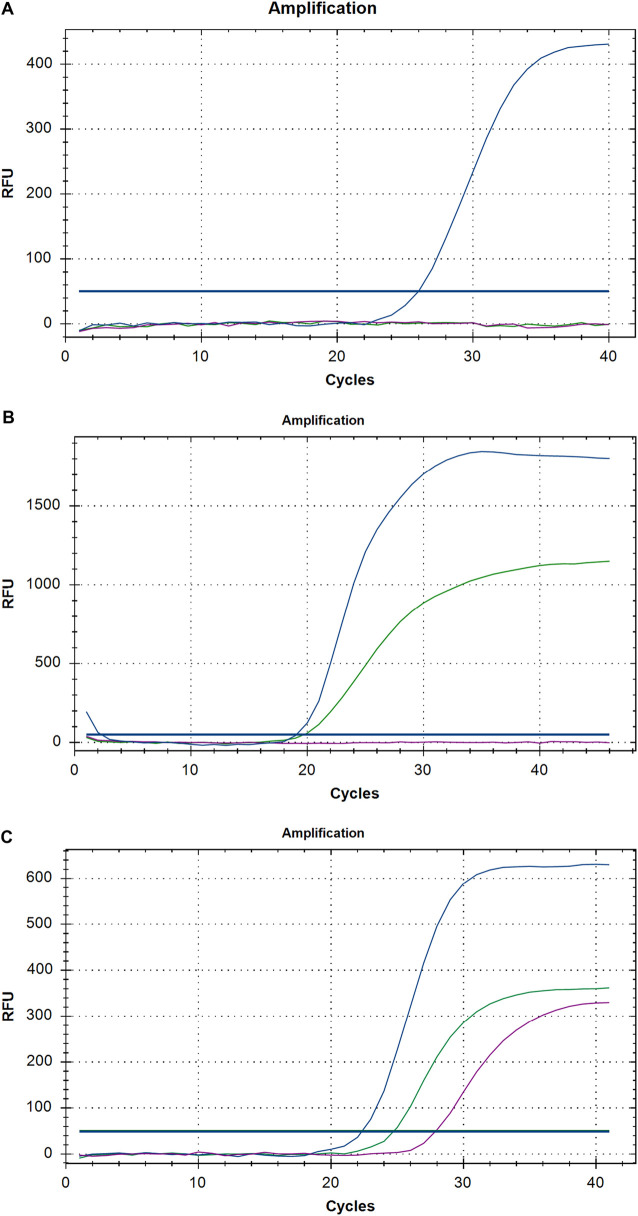
A representative graph of Real-time PCR exhibiting **(A)** United Kingdom, **(B)** South African and Brazilian **(C)** other then VOC of SARS-CoV-2.

All replicating viruses including SARS-CoV-2 continuously accumulate genetic mutations that persist due to natural selection. Some of these mutations may bring functional changes resulting in an increased intensity of infection and rate of proliferation of the virus. We firstly investigate mutations related to SARS-CoV-2 VOC by RT-PCR method from SARS-CoV-2 positive samples, divided in two different quarters, of three different labs in Karachi. We identified that the total tests conducted in group I (first quarter) were 16,964 while in group II (Second quarter) were 46,041 respectively. Comparing the positivity rate of SARS-CoV-2 infection in two groups the average positivity ratio in group I was 1.53% which is significantly lower than the positivity ratio of 7.60% of group II respectively (*p* < 0.05) (Table 2). In the group II variants of SARS-CoV-2 other than VOC were predominantly enriched (46.3%), however on comparing it with group I a significant difference was noted (*p* < 0.05). Further, we identified none of the VOC in Group I. While comparing it with group II a significant difference was noted. The results showed B.1.1.7 (United Kingdom), B.1.351 (South African), and P.1 (Brazilian) VOC at higher frequencies. About 26.96% of samples came out to be the UK variant in group II exhibiting mutation in ∆69–70 as well as ∆3675–3766. Moreover, we report that another 26.67% positive samples exhibit single target failure ∆3675-3766-del which implied the presence of South African and Brazilian variants. While 1623 (46.30%) samples did not show any failure of target and represent other lineages. Amplification curves exhibiting SARS-CoV-2 variants of concern, detected by target failures using a multiplex RT-qPCR, are shown in Figure 1.

**TABLE 2:** Scenario of SARS-CoV-2 B.1.1.7, B.1.351, and P.1 variants of concern in Karachi, Pakistan.

	Period selected for analysis	Total #of samples received for RT-qPCR testing	Total # of PCR positive samples	Percentage of PCR positive samples (%)	Scenario of SARS-CoV-2 B.1.1.7, B.1.351, and P.1 variants of concern in Karachi		
					B.1.1.7	B.1351 and P.1	Other lineages
Group I	August	4,638	90	1.94	0	0	90
	September	5,833	69	1.182	0	0	69
	October	6,493	102	1.57	0	0	102
	Total	16,964	261	1.538	0	0	261
Group II	November	9,233	610	6.60	164 (26.88%)	68 (11.14%)	378 (61.96%)
	December	19,299	1,682	8.71	474 (28.18%)	484 (28.77%)	724 (43.04%)
	January	17,509	1,209	6.90	326 (26.96%)	362 (29.94%)	521 (43.09%)
	Total	46,041	3,501^*^	7.60	944 (26.96%)	934 (26.67%)	1623 (46.3%)

We then investigate the association between age and gender among confirmed cases in two groups. It is reported that the mean age in group I was 38.01 (±16.403), and in group II found to be 34.05 (±15.219) (Table 3). However, the correlation of age between the groups is not statistically significant (*p* > 0.5). It is important to note that since the beginning of the COVID-19 pandemic it appeared clearly from the first Chinese reports that a higher proportion of men get infected as compared to women. A similar pattern was also observed in Italy in a significantly older population when compared to the Chinese one (Jin et al., 2020). In the present study, we examine the effect of the SARS-CoV-2 VOC on gender difference. It was found that in group I where no VOC was reported 58 out of 261 positive samples were women (22.22%), while in group II an increased number i. e, 950 out of 3,501 were women (27.01%). Comparison of gender exhibits statistically significant difference among groups (*p* < 0.5).

**TABLE 3:** Demography of sample population and the variants of concern in Karachi, Pakistan.

	Total #of samples for RT-qPCR testing	Total # of PCR positive samples	Positivity percentage (%)	Male to female ratio		Mean age
				Male (%)	Female	
Group I	16,964	261	1.538	77.85	22.15%	38.01 (±16.403)
Group II	46,041	3501	7.60	72.99	27.01%^*^	34.05 (±15.219)

## Discussion

The PCR tests for SARS-CoV-2 detection in the United Kingdom during September 2020 have shown an unusual SARS-CoV-2 strain that prevents amplification of the spike gene, also known as spike gene target failure (SGTF) in a commercially available kit (Wang et al., 2021). The failure to detect the spike gene is attributed to a ∆69/70 HV deletion as in the lineage B.1.1.7, commonly known as the UK variant. Moreover, the P.1 lineage in Brazil and the B.1.351 lineage described in South Africa share a common set of mutations – ORF1a deletion 3675–3677 SGTF (Tegally et al., 2020). Therefore, a sample with ∆69/70 HV deletion is not enough for discriminative detection of B.1.1.7 from other variants of concern i. e, B.1.351; South African and P.1; Brazilian VOC, which does not possess ∆69/70 HV deletion. Therefore, a multiplex PCR assay reported earlier targeting ∆3675–3677 SGTF deletion in ORF 1a gene along with ∆69/70 HV deletion is used in the present study to discriminate B.1.1.7 from B.1.351 and P.1 variants of SARS-CoV-2 (Vogels et al., 2021). We have used CDC_N1 primer and probe as a control to ensure that target failures were due to these mutations. In resource-limited countries like Pakistan, the present study can therefore be helpful to rapidly scale up the prediction of SARS-CoV-2 variants.

The SARS-CoV-2 VOC are the cause of apprehension globally, not only because of their ability to transmit rapidly but also because of antigenic changes due to multiple mutations in the spike protein region unfavorable to monoclonal antibody (mAB) therapies and protection by vaccines (Faria et al., 2021; Naveca et al., 2021). The variant P.1 is spreading at a rapid pace in Brazil and spreading globally. N501Y is the common mutation shared between VOC. This particular mutation may result in augmented binding to ACE231 and exhibit no significant escape value from emergency use authorized mAB therapies and vaccines (Voloch et al., 2021). However, the E484K mutation present in B.1.351 and P.1 variants has been attributed to impairing many mABs and vaccines under development and is also responsible for re-infection (Tegally et al., 2021). This threat has been augmented by the recent observation from the Novavax vaccine trial in South Africa which showed that exposure to B.1.351 in placebo recipients’ exhibit being un-protective against prior SARS-CoV-2 infection (Naveca et al., 2021; Sabino et al., 2021; Voloch et al., 2021). Even in Brazil, the same mutation in P.1 is reported to be responsible for the spreading of the virus in a population that was already 76% seropositive due to past infection (Sabino et al., 2021). This highlights the importance of this study of mapping viral variants by RT-qPCR which could be adopted by limited-resource countries worldwide. This mapping would be helpful in the effective development of therapies and vaccines targeting antigenically distinct epitopes.

We demonstrate the presence of UK (B.1.1.7; 27.04%), and South African and Brazilian (B.1.351 and P.1; 29.94%) SARS-CoV-2 VOC from the positive samples collected in group II. The trend of emergence of VOC in the United Kingdom, South Africa, and Brazil, the respective countries where these variants were first reported, is in agreement with the present study (Babb de Villiers et al., 2021). Strikingly, the positive samples archived before November 2020 (group I) did not exhibit VOC. It is noteworthy that in group I the positivity ratio was 1.538% which later increased to 7.60% in group II. This clearly indicates the importation of cases from countries where these viral strains were spreading before and during November 2020. The spread of the pandemic globally provides an opportunity for variable strain types to thrive. One of the reasons for this spread could be the unmonitored international flights and lack of quarantine on arrival in Pakistan from any country, which lead to a rapid introduction of variants of concern in Pakistan (Mukry et al., 2021; Umair et al., 2021). Pakistan is in its second wave which could not be truncated due to variant entry, which prevented a decline to baseline levels following the first wave. The second peak of the second wave is labeled as the third wave, and has affected the Prime minister, First Lady, and President of Pakistan. Not only the notables of the country have been affected in this notorious second wave, but our results also revealed that the positivity ratio in women has significantly increased from 22.15 to 27.01%. This clearly indicates that VOC are more transmittable.

It can be deduced from the current study that diffusion of VOC B.1.17, B.1351, and P.1 has epidemiological consequences. For instance, in Pakistan during the first quarter from August 2020 to October 2020 when none of the VOC were found, the number of deaths attributed to SARS-CoV-2 were reported to be 853, while after identification of VOC in significant numbers in the second quarter from November 2020 to January 2021, the number of deaths by COVID-19 increased to 4,860 (www.covid.gov.pk/stats/pakistan access date; 23-09-2021). A similar pattern was observed in Sindh where the number of SARS-CoV-2 related deaths reported in Group I (no variants) was 407 and in Group II (VOC in significant numbers) the number raised to 1,369. The effect of VOC in Sindh can further be seen by the number of critically ill patients which in October 2020 (Group I) was 179 including 30 on ventilators. While in January (Group II) after the appearance of VOC a staggering difference could be noted, as there were now 768 critically ill patients including 76 on ventilators were reported (www.sindhhealth.gov.pk access date; 23-09-2021). The increase in numbers of deaths and critically ill patients in Group II could be attributed to the diffusion of VOC (COVID, 2021).

At present, the significance of the surveillance of variants has increased globally as the vaccination jabs have started and countries race to vaccinate. Limited-resource countries are lagging behind and with the emergence of these variants in these countries it is of high possibility that an escape variant could evolve which could hinder the vaccination process globally. The vaccination program in Pakistan is slow despite an increase in SARS-CoV-2 infection numbers. There is a need to expedite vaccination in limited-resource countries including Pakistan through the COVAX program or any other channel as ‘no one is safe until everyone is safe’. If they remain unvaccinated, limited-resource countries might act as the breeding ground for the variants of the virus that could be detrimental to global efforts for ending the pandemic.

The striking part of our study is the presence of a variant with ∆3675–3677 SGTF deletion in the ORF 1a gene which implied the presence of the South African and Brazilian variants in Pakistan. The most remarkable part of the present study is that the South African and Brazilian variants are greater in their numbers in December 2020 and January 2021 as compared to the UK variant, while it was showed that SARS-CoV-2 with lineages other than the VOC are fewer in numbers in positive samples collected during and after November 2020. The emergence of the United Kingdom, South African, and Brazilian variants calls for strict measures to curb the spread of SARS-CoV-2. The lucidity in this dark situation can only be attained by the arrangement of non-pharmaceutical interventions and scaling up-of vaccination programs, which could lead to fewer infections, and in turn lead to fewer variants. Both strategies are important until population immunity is achieved globally.

The current pandemic should nudge the developed countries that infectious diseases have a profound impact on lives and economies, and rapid distribution and implementation of useful vaccines against these infections should remain a priority. Global cooperation to make certain a just and responsive reaction to local contexts is necessary on the complex trail forward to ending the COVID-19 pandemic.

## Data Availability

The datasets presented in this study can be found in online repositories. The names of the repository/repositories and accession number(s) can be found in the article/Supplementary Material.
