# Supplementary figures and images for: SARS-CoV-2 UK, South African and Brazilian Variants in Karachi- Pakistan
Source: Front Mol Biosci. 2021 Oct 25;8:724208. doi: 10.3389/fmolb.2021.724208 (PMC8572847; doi:10.3389/fmolb.2021.724208)

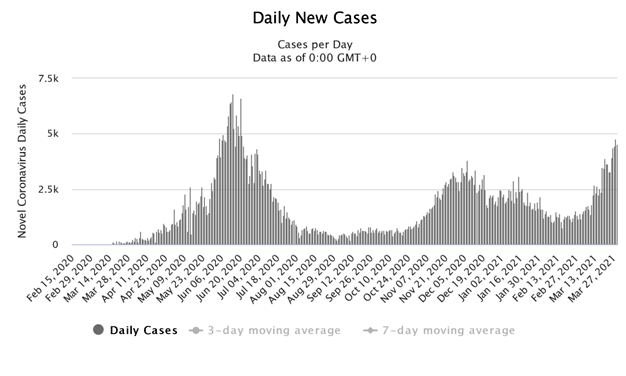

Supplement: Supplementary file 1 [file Image1.TIF]
